# An update on oral manifestations of systemic disorders in dogs and cats

**DOI:** 10.3389/fvets.2024.1511971

**Published:** 2025-01-06

**Authors:** Claire Dosenberry, Boaz Arzi, Carrie Palm, Natalia Vapniarsky, Maria Soltero-Rivera

**Affiliations:** ^1^Dixboro Veterinary Dental Clinic, Ann Arbor, MI, United States; ^2^School of Veterinary Medicine, Veterinary Surgical and Radiological Sciences, University of California, Davis, Davis, CA, United States; ^3^School of Veterinary Medicine, Veterinary Medicine and Epidemiology, University of California, Davis, Davis, CA, United States; ^4^School of Veterinary Medicine, Department of Pathology, Microbiology, and Immunology, University of California, Davis, Davis, CA, United States

**Keywords:** oral medicine, feline, canine, systemic disorders, dentistry

## Abstract

Oral lesions are common in dogs and cats, and determining the underlying etiology of these lesions can be challenging. A wide range of systemic ailments may lead to lesions in the oral cavity, including immune-mediated diseases, adverse drug reactions, viral and bacterial infections, and metabolic and autoimmune diseases. A complete history and thorough physical examination (including a fundic examination) should be obtained in affected patients. It is critical to perform a detailed oral examination, which in some patients may need to be performed under sedation or general anesthesia. Tailored diagnostic plans and a multidisciplinary approach are necessary to fully characterize co-morbid disorders in affected patients. This narrative review aims to aid veterinarians in recognizing oral manifestations of systemic disorders based on the most recent reports and available research.

## Introduction

Systemic disorders in dogs and cats may manifest as focal, multifocal, or generalized lesions in the oral cavity. Working knowledge of the clinical presentation of these diseases can aid in reaching a definitive diagnosis. Both general practitioners and specialists can benefit from understanding and recognizing the lesions they may encounter in the oral cavity to determine if the animal is suffering from a primary oral condition or if a more comprehensive and/or systemic medical disorder is present. Recognizing when oral lesions manifest more global systemic disease is crucial, as treating the underlying systemic disease may alleviate the oral symptoms and improve the patient’s quality of life.

Oral lesions are common but may go unnoticed if the patient is not exhibiting obvious clinical signs of oral pain, if the patient is not tolerant of an oral examination, or if a complete examination is not performed. A thorough history is pivotal to determine what existing co-morbidities, medications, or infectious agents the patient may have been exposed to. Detecting additional systemic clinical signs the patient may be experiencing is also crucial.

Limited knowledge of the oral manifestations of systemic diseases in dogs and cats exists (1). This review aims to update the existing literature and assist clinicians in formulating differential causes in the process of identifying if oral lesions represent primary or systemic processes.

## The oral exam in the healthy patient

Recognizing healthy, normal anatomy in the dog and cat during routine oral examinations is important. The teeth should be covered by a thin enamel layer and appear smooth and white. The gingiva should be pink or pigmented, smooth, and wrapped around the base of the crown. The rest of the oral cavity is lined by mucosa, which is moist, pink, or pigmented in healthy animals. The mucosa should be smooth except for the dorsal tongue papillae, the hard palate rugae, and areas with gingival stippling, which are keratinized areas due to their role in mastication.

The oral mucosa can be divided into two other groups. Lining mucosa makes up 60% of the oral mucosa and is non-keratinized. The lining mucosa comprises the sublingual, alveolar, buccal, and soft palatal mucosa. Finally, the specialized mucosa of the dorsal tongue makes up 15% of the oral mucosa and contains taste buds (2). The mouth should be free from foul odors, and saliva should be clear in color.

Abnormalities of the oral mucosa due to systemic diseases can be subdivided into endocrine and metabolic, vesiculobullous and autoimmune, infectious, hematologic, and miscellaneous disorders, and that is the structure this manuscript will follow.

## Oral manifestations of endocrine and metabolic diseases

### Congenital hypothyroidism

**Figure 1:**
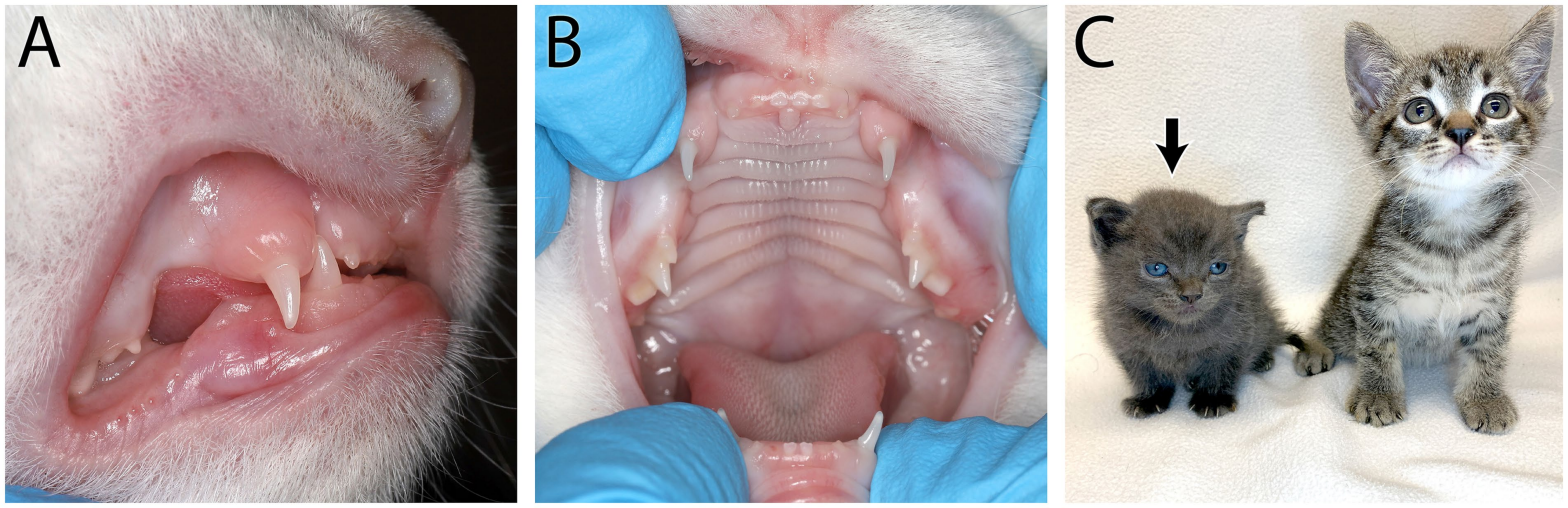
Feline congenital hypothyroidism in a 6-week-old intact female domestic short hair kitten that presented for lethargy and mental dullness. On physical examination it was noted she had incomplete deciduous dentition **(A)** and partially erupted deciduous dentition **(B)**. Short stature and a broad face can be seen on the affected kitten (black arrow) compared to a normal kitten of the same age **(C)**. Congenital hypothyroidism was confirmed with a thyroid panel showing low total thyroid hormone (T4), free T4 (FT4) and thyroid stimulating hormone (TSH).

Congenital hypothyroidism in cats is a rare disease with an autosomal recessive mode of inheritance. If diagnosed at a young age, this disease can be successfully treated with thyroid supplementation (3). Kittens affected with congenital hypothyroidism have disproportionate dwarfism manifested with a broad head and short neck phenotype. Examination of the oral cavity may reveal partially erupted or unerupted permanent dentition, covered with a thickened fibrous gingiva (4, 5) and persistence of deciduous teeth (Figure 1). If suspected, hypothyroidism can be confirmed by a thyroid panel that includes the total thyroid hormone (T4), free T4 (FT4), and thyroid stimulating hormone (TSH) (4).

### Hyperparathyroidism

Hyperparathyroidism is characterized by elevated blood parathyroid hormone levels that can result in hypercalcemia and other systemic consequences (5). Primary hyperparathyroidism occurs when a functional parathyroid gland tumor, usually a functional adenoma, autonomously produces excess parathyroid hormone (PTH). Excessive secretion of PTH results in persistent hypercalcemia and hypophosphatemia (5). Vomiting, polyuria, polydipsia, lethargy, and weakness represent clinical signs of hypercalcemia and should be considered in conjunction with oral lesions. In secondary hyperparathyroidism, which results from nutritional deficiencies or chronic kidney disease, plasma total calcium concentrations are either normal or slightly decreased, and, depending on the cause, plasma phosphorus concentrations are either normal or increased (5). Secondary renal hyperparathyroidism occurs in almost all dogs and cats with advanced and end stage kidney disease (6). Nutritional hyperparathyroidism is rarely seen in domestic animals because commercial diets balance calcium and phosphorus adequately.

However, obtaining a detailed diet history for every patient is still critical with the popularity of home-cooked diets that can be nutritionally unbalanced. Regardless of the primary cause of hyperparathyroidism, the action of excess PTH is calcium and phosphorus release from the bone by osteoclastic resorption, leading to osteopenia.

**Figure 2:**
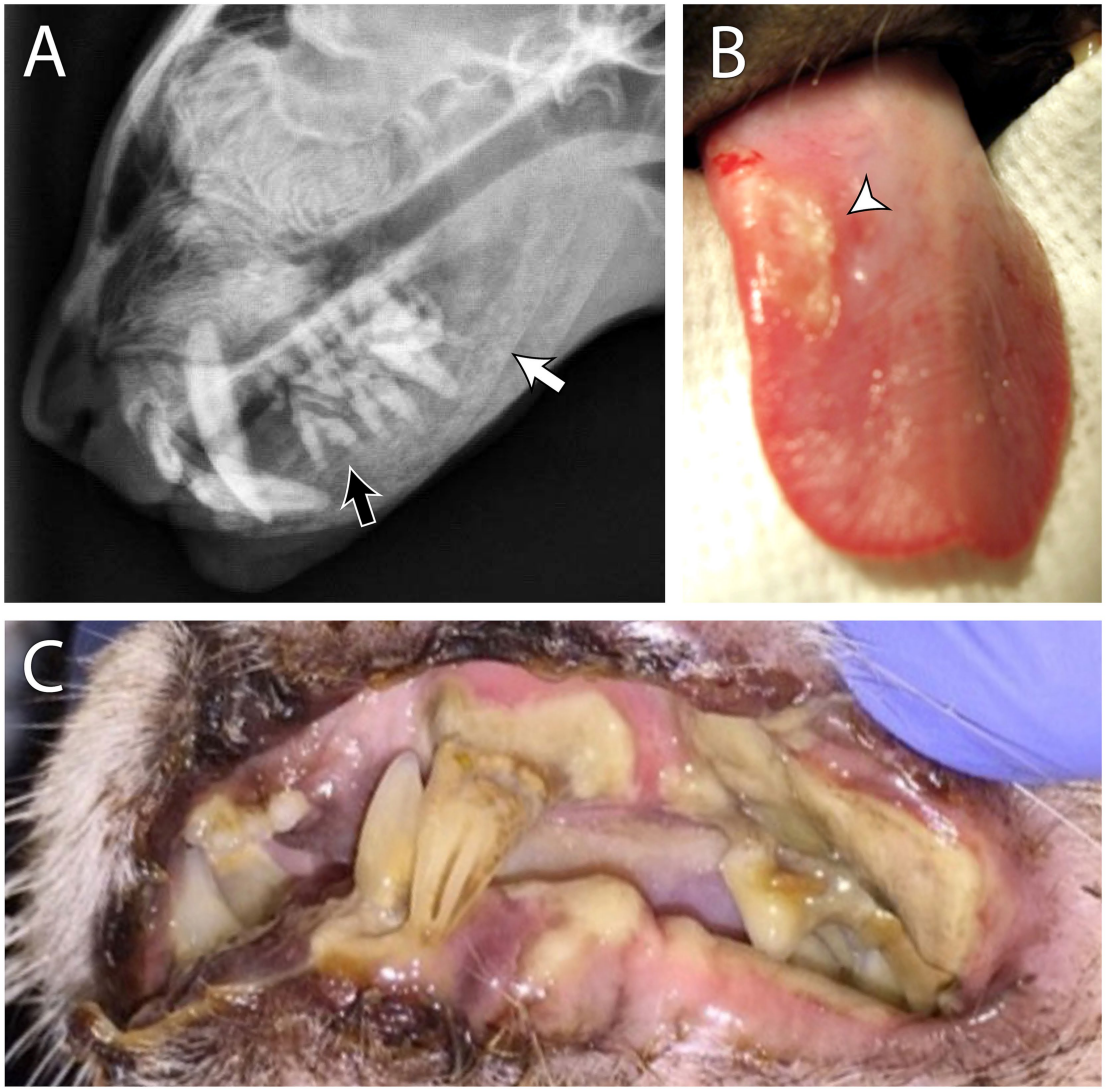
Secondary renal hyperparathyroidism in a 4 year old male neutered domestic short hair cat. The patient had a history of dilute urine, inappropriate urination, weight loss, lethargy, and worsening hyporexia. Blood work revealed azotemia, hyperphosphatemia, anemia, and isosthenuria. Skull radiographs **(A)** show a ground glass appearance on the mandibular and maxillary bones, indicating osteopenia (white arrow). The lamina dura is also absent around the tooth roots (black arrow). These findings are consistent with a diagnosis of “rubber jaw.” Examples of uremic ulcers of the tongue **(B)** and the oral mucosa **(C)** from different patients are (also) shown.

Oral manifestations of osteopenia include demineralization of the jaw bones with resultant painful mastication and dysphagia, teeth mobility, and gingivitis (7). In affected patients, the alveolar and cancellous bone of the mandible and maxilla are more prone to demineralization than other sites, such as long bones, and oral manifestations are therefore present before clinical signs such as lameness. As demineralized tissue is replaced by fibrous tissue, affected bones lose stability and result in a condition known as “fibrous osteodystrophy” or “rubber jaw.” Gross enlargement of the jaws is uncommon but has been documented in young dogs with advanced congenital kidney disease due to fibrous replacement of demineralized bone, as above (8, 9). The effects of osteopenia in the oral cavity, particularly mobile teeth, can easily be mistaken for severe periodontal disease, often mimicking periodontal-endodontic lesions (10). However, patients affected by hyperparathyroidism typically exhibit minimal to mild gingivitis and normal periodontal probing depths. Radiographically, the absence of the lamina dura, widened periodontal ligament space in the absence of periodontal attachment loss, and a ground glass appearance of the trabecular bone (Figure 2) should make a clinician suspicious for hyperparathyroidism and not primary periodontal disease. Secondary hyperparathyroidism due to renal disease should be suspected if uremic ulcers are detected in addition to osteopenia (Figure 2).

The ulcers are formed due to uremic vasculopathy that develops due to urea degradation into ammonia with its direct damage to endothelial cells (11). To determine if teeth truly need to be extracted in hyperparathryoid patients, extractions should be delayed until osteopenia is resolved or improved with medical management. Hence, if suspected, hyperparathyroidism should be ruled out by obtaining a complete history, physical examination, complete blood count, biochemical profile, and diagnostic imaging.

### Acromegaly

Acromegaly is a functional disturbance of growth resulting from hypersecretion of growth hormone (GH). This syndrome has been reported in dogs and cats (12). Acromegaly in cats is usually caused by excessive production of GH by a functional acidophil (somatotroph) adenoma in the pars distalis of the pituitary gland (13, 14). Excessive GH results in the downregulation of insulin receptors and resistance to insulin action at the cellular level. Somatotroph adenomas in cats are considered to be a predisposing cause of insulin-resistant diabetes mellitus, which is difficult to control in these patients (14). Recent studies suggest that acromegaly may be present in as many as 30% of insulin-resistant diabetic cats and should be considered a differential diagnosis, especially when associated clinical features are present (14, 15). In dogs, acromegaly usually results from functional GH-producing tumors in organs other than the pituitary (5).

Clinically, acromegaly is characterized by an overgrowth of connective tissue, increased appositional growth of bones, coarsening of facial features, gingival hyperplasia, separation of teeth, macroglossia, and visceral organ enlargement. In cats, reports describe enlargement of the jaws and calvarium, resulting in a broad face and mandibular prognathism (13). Excessive soft tissue growth leads to an enlarged tongue and thickening of the soft palate and pharyngeal tissues, which can cause respiratory stridor in up to 50% of affected cats (15). The uncontrolled growth of the flat bones of the skull and mandible seen in this disease results in increased interdental spaces (12). The proliferation of joint cartilage may cause degenerative arthropathy. Kidney disease also may develop as a result of periglomerular fibrosis. Diagnosis of acromegaly requires a combination of tests, including measurements of IGF-1 (Insulin growth factor-1), GH, and intracranial imaging (14).

### Diabetes mellitus

Diabetes mellitus (DM) occurs in patients with increased cellular resistance to insulin and/or decreased production and secretion of insulin by the pancreas. In humans, the oral manifestations of DM have been extensively researched and documented. The most prevalent clinical signs in humans include salivary dysfunction (xerostomia) and increased oral infections that may result in gingivitis, periodontitis, and oral candidiasis (16). An amplified inflammatory response to the normal flora in the oral cavity is thought to precipitate periodontitis in patients with diabetes (17). Veterinary literature investigating oral manifestations of DM in dogs and cats is limited to a single case report of a diabetic cat with root retention after dental surgery that caused subsequent sepsis and a severe systemic inflammatory response (18). The author’s clinical experience indicates that patients affected with diabetes mellitus are often presented with moderate to severe periodontitis and that dental cleaning and removal of bacterial biofilm facilitate subsequent glycemic control. It is likely that significant oral inflammation and periodontitis causes insulin resistance in some patients with DM (19). As such, thorough oral evaluation and comprehensive periodontal treatment and extraction of periodontically and endodontically affected teeth may help in the overall management of DM.

## Oral manifestations of immune-mediated, vesiculobullous and autoimmune diseases

A summary of the most common lesions locations for immune-mediated, vesiculobullous and autoimmune diseases can be found in Table 1.

**Table 1:** Immune-mediated, vesiculobullous and autoimmune diseases affecting the oral mucosa, mucocutaneous junctions, haired skin, or combinations thereof in dogs and cats.

Vesiculobullous, autoimmune, and immune-mediated diseases	Haired skin	Mucocutaneous junctions	Oral mucosa
Discoid Lupus Erythematosus	X		
Systemic Lupus Erythematosus			X
Erythema Multiforme	X	X	X
Pemphigus Vulgaris	X		X
Pemphigus Foliaceus	X		
Bullous Pemphigoid	X		
Epidermolysis Bullosa Acquisita	X	X	X
Mucous Membrane Pemphigoid		X	X (primarily)
Paraneoplastic Pemphigus	X		X (dogs)

The table highlights the specific areas impacted by each condition, aiding in the identification and diagnosis of lesions in different anatomical regions.

### Erythema multiforme

Erythema multiforme (EM) is generally an entity within a spectrum of cytotoxic epidermal diseases that also include Stevens-Johnsons Syndrome (SJS; a more severe form of EM) and toxic epidermal necrolysis (TEN; the most severe form) (20). There is no uniform consensus on the classification of these diseases, and the clinician should be aware that histopathological features cannot distinguish EM/SJS/TEN reliably, since they are very similar and differ mainly in clinical severity (21). There is also uncertainty regarding what triggers these conditions. Medications, viral, and bacterial infections were documented along with idiopathic cases, in which no such associations were found. Animals with SJS/TEN are usually presented as very sick and febrile, with leukocytosis, neutrophilia, hypergammaglobulinemia, and other serum biochemical abnormalities (22). In humans, SJS and TEN cases are separated based on the degree to which the surface area is affected, with TEN represented by skin reaction over 30% of the body surface area. The skin and mucosal lesions are typically macular erythema that progress to blisters and ulcers with exudation. Nikolsky’s sign (where epidermis sloughs with minimal shear force applied) may be present adjacent to the affected areas (23). Several biopsy samples together with the application of algorithms for drug causality, such as the Assessment of Drug Causality in Epidermal Necrolysis (ALDEN), can be helpful when evaluating suspected cases of TEN (24). In humans and animals, immediate emergency attention and aggressive supportive treatment are warranted.

Erythema multiforme is a less severe rare vesiculobullous and ulcerative skin condition that affects mucocutaneous junctions, including those in the oral cavity. Patients with EM-related oral lesions develop severe ulcerative stomatitis and glossitis (25). Based on the limited research available, approximately 30% of dogs with EM may have oral involvement (26), and there have been no reported cases of EM that are limited only to the oral cavity (27). Commonly affected areas of skin in the body with EM classically include the ventral abdomen and axillae, mucocutaneous junctions, footpads, and pinnae. EM is thought to be a hypersensitivity reaction, and the most common reported trigger in the dog has been drug therapy, including, but not limited to, penicillins and sulfonamides (28). Other suspected triggers include adverse food reactions, infection (i.e., bacterial skin infection, parvovirus), or neoplasia (i.e., splenic sarcoma, thymoma) (29). Chronic EM in old dogs is often idiopathic (30). Definitive diagnosis of EM can be challenging but is typically based on a compatible history of a known trigger, compatible clinical findings, and histopathological findings that include lymphohistiocytic lichenoid infiltrates and keratinocyte necrosis with lymphocytic satellitosis at all levels of the epidermis (25). Depending on the severity, important clinical differentials of this condition include SJS, TEN, thermal or caustic burns, epitheliotropic lymphoma, and other immune-mediated dermatoses.

### Pemphigus

Pemphigus is a group of autoimmune bullous skin diseases affecting multiple species, including dogs and cats. Pemphigus Vulgaris (PV), Pemphigus Foliaceous (PF), and Paraneoplastic Pemphigus (PNP) result from an autoimmune attack on desmosomes, leading to the breakdown of cell-to-cell adhesion in the mucosa and epidermis through acantholysis (31). When these connections are destroyed, the epithelial tissue layers separate, forming pustules, vesicles, bullae, erosions, and ulcers on the mucosa, haired skin, and mucocutaneous junctions.

#### Pemphigus vulgaris

**Figure 3:**
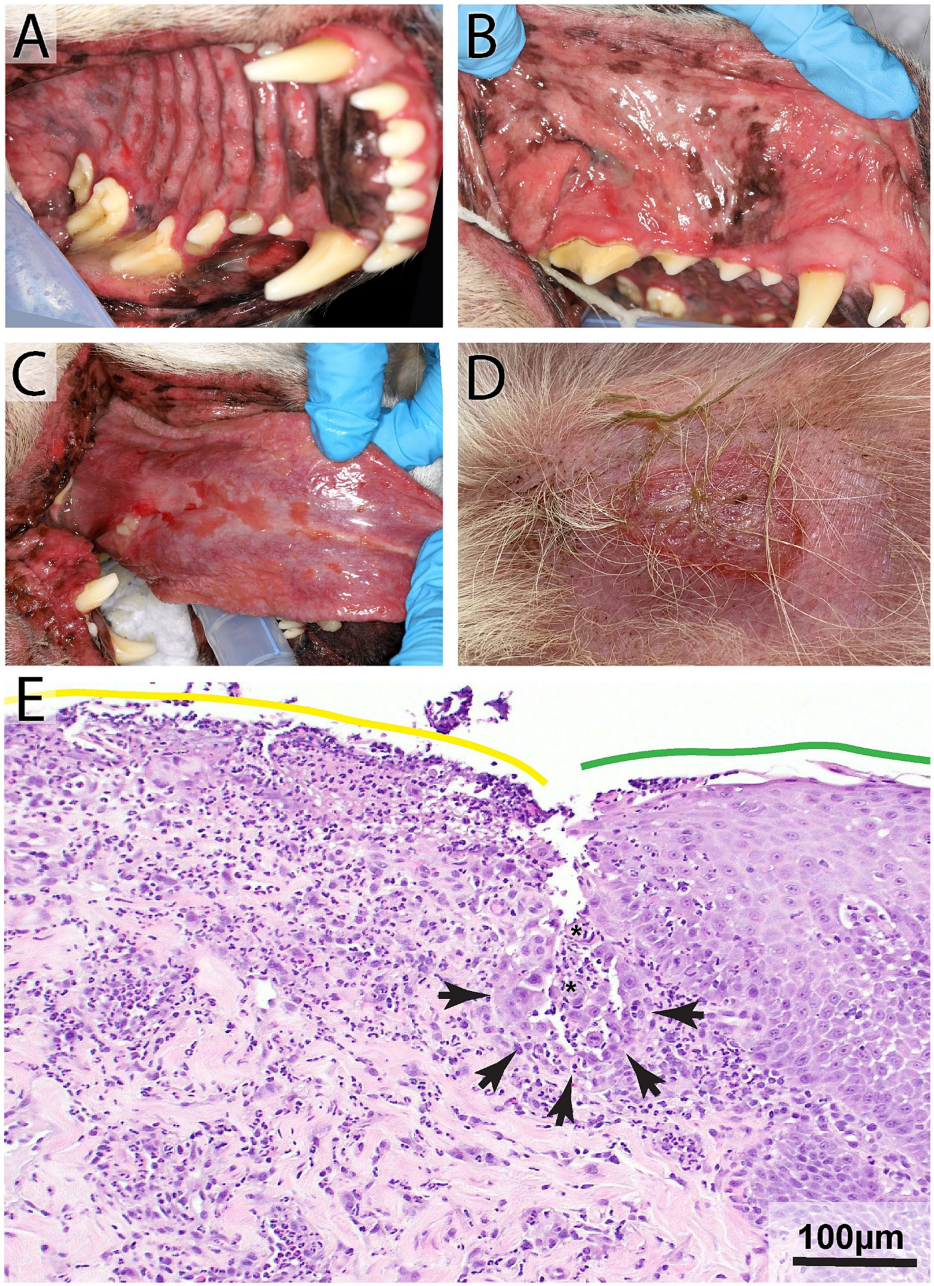
Pemphigus vulgaris in an 8-year-old male neutered Labrador Retriever with a 10-day history of lip-smacking, gagging, halitosis, and thick, ropey saliva. Physical examination revealed moderate to severe ulcerations on the hard and soft palatal mucosa **(A)**, buccal mucosa **(B)**, lingual and sublingual mucosa **(C)**, mucocutaneous junctions, and gingiva. Lesions were also found on the patient’s skin **(D)**. Histopathology highlighting the junction of ulcerated (yellow line) and intact (green line) palatal epithelium **(E)**. Note supra basilar clefting (black arrowheads) lined by a single layer of keratinocytes that exhibit tomb-stoning and acantholytic cells (asterisks) H&E, Bar = 100 μm.

Desmoglein 3 is the targeted protein in mucosal and mucocutaneous dominant PV in humans and dogs (31–34). Autoantibodies against desmoglein 1 may also be detected if skin lesions are present (32). PV is considered rare in dogs and cats, yet is the most likely type of pemphigus to manifest with oral lesions. It is considered a life-threatening disease due to a complete loss of the epithelial barrier. Based on previous studies, over 90% of dogs with PV had oral lesions, and in 6% of dogs the oral cavity was the only location affected (31). Lesions are most likely found in areas of keratinized oral mucosa, including the hard palate, gingiva, and dorsal tongue (25) (Figure 3). Common oral lesions include flaccid vesicles and bullae, ulcerations, and erosions. The vesicles are fragile and are not always noted on physical examination due to having already ruptured prior to presentation (32). These patients will often have hypersalivation, halitosis, and lip-smacking due to oral pain secondary to the ulcers. The defining histopathological feature is suprabasilar clefting with only a single layer of rounded basal cells remaining (‘tombstones”) and acantholysis (31). The manifestation of oral lesions in PV contrasts with PF, which is almost exclusively associated with dermal lesions (32).

#### Paraneoplastic pemphigus

Paraneoplastic Pemphigus (PNP) is a sporadic autoimmune bullous skin disease associated with underlying neoplasia. There have only been a few case reports in dogs and one in cats (35–37). Unlike humans and dogs, the cat had no oral lesions (36). The most predominant clinical feature in humans with PNP is severe stomatitis. This stomatitis is typically the first clinical sign to appear and is characterized by diffuse ulcerations that span from the oropharynx to the lips (38). In all three case reports, dogs with PNP had extensive oral ulcerations and erosions, with the tongue and gingiva most commonly affected (31). In both dogs and humans, histologic features of PNP combine features of PV and EM, including suprabasal acantholysis, lymphohistiocytic lichenoid infiltrates, and apoptosis of keratinocytes in the epithelium with lymphocyte satellitosis (25). A full diagnostic work-up to rule out malignancies that have been associated with PNP (i.e., splenic sarcoma and thymoma), including a thorough physical examination and abdominal and thoracic imaging, should be performed when histology results show features consistent with both PV and EM (35, 37).

### Lupus erythematosus

Lupus Erythematosus (LE) occurs in various forms, and each specific form differs by the tissues involved and the localization and distribution of lesions. This spectrum of inflammatory disorders ranges from mild skin-limited conditions to life-threatening systemic disease (systemic LE). The classification of Lupus erythematosus is complex and includes discoid lupus (DLE), systemic lupus (SLE) and variants of cutaneous lupus erythematosus (CLE) (39). The forms most commonly affecting the oral cavity in dogs are DLE and SLE. Classically, DLE is presented with depigmentation, erythema, and scaling frequently restricted to the face, particularly the nasal planum, symmetrically around the eyes, pinnae, lips, and vulva. The lesions may progress from erythema to erosions, ulcerations and scars. Oral lesions, manifested as focal ulcers, are rare in discoid lupus erythematosus (DLE) and mucocutaneous lupus erythematosus (MCLE), with perioral lesions being more common than intraoral lesions (25). In one study of 21 dogs with MCLE, only three showed mild ulcerations in the oral cavity on the gingiva or palate (40). DLE is most commonly diagnosed in the skin, and lesions are aggravated by sun exposure. Given this, cutaneous lesions typically appear before oral lesions (25). Thus, the timing of lesion development, with skin lesions preceding oral lesions, may help in DLE diagnosis when other associated features are present. With SLE, polyarthritis, thrombocytopenia, fever of unknown origin, anemia, stomatitis, and glomerulonephritis are common manifestations, while skin lesions are actually rare (20).

### Autoimmune subepidermal blistering diseases

Autoimmune subepidermal blistering diseases are rare chronic skin conditions in humans and companion animals. In this group of diseases, vesicles and bullae form due to antibodies targeting the adhesions between the epithelium and the basement membrane; these lesions rupture to form deep erosions and ulcers (41, 42). AISBD in dogs and cats include mucous membrane pemphigoid, epidermolysis bullosa aquisita, and bullous pemphigoid. These can be diagnosed via recognition of histopathologic features, detailed medical history, and distribution of lesions.

#### Mucous membrane pemphigoid

MMP is the most common AISBD seen in dogs, accounting for 48% of cases, with German shepherd dogs overrepresented (41). Autoantibodies target various dermal and basal epidermal epitopes such as collagen XVII, BP230, integrins, laminins, collagen IV, VII in the basement membrane, among other proteins (43, 44). Vesicles and erosions are found on the mucous membranes and mucocutaneous junctions and the lesions were symmetrical in almost 90% of 16 patients in one study (45). The most common intraoral locations are the gingiva, hard and soft palate, and the tongue (41). The nasal planum can be the second most common site to be affected and may be the earliest one to manifest lesions (45). In one study, approximately half of the dogs were reported to have other clinical signs associated with the oral cavity, including malodor, pain when eating, and hypersalivation (45). The skin of dogs with MMP is not often affected, but lesions may be found on mucocutaneous junctions on other parts of the body, including the eyes, nose, genitals, and anus (25). MMP has been described in four cats, and lesion type and distribution are similar to those of dogs (41). Histologically, large subepidermal clefts devoid of inflammatory cells at the margin of an ulcer is a typical feature. A band of fibrosis can be present in the superficial dermis (45). To distinguish this entity from pemphigus vulgaris, epidermolysis bullosa, and vesicular lupus erythematosus histologically is challenging, especially at the ulceration stage.

#### Epidermolysis bullosa aquisita

Epidermolysis bullosa aquisita is recognized in humans and dogs and is a non-genetic form of severe blistering and erosions of the oral mucosa, mucocutaneous junctions, and haired skin. Affected dogs are typically young, with 45% developing lesions prior to 1 year of age. Great Danes and German shorthaired pointers are overrepresented. The oral cavity is involved in 92% of cases, and these patients are often systemically ill with fever, enlarged lymph nodes, and inappetence (41). Histological features include epidermal separation with or without basal cell degeneration. Subepidermal vesicles that form as a result may contain red blood cells. Inflammation is usually scarce in the early lesion but may become more pronounced with ulceration characterized by a neutrophilic infiltrate (46).

#### Bullous pemphigoid

Bullous pemphigoid (BP) has been described rarely in dogs and cats and is the most common autoimmune subepidermal blistering disease in humans (47). Lesions in BP are primarily found in haired skin. Mucosal involvement is mild compared to the other AISBD’s and includes vesicles and erosions/ulcers (41).

## Oral manifestations of infectious diseases

### Feline calicivirus

**Figure 4:**
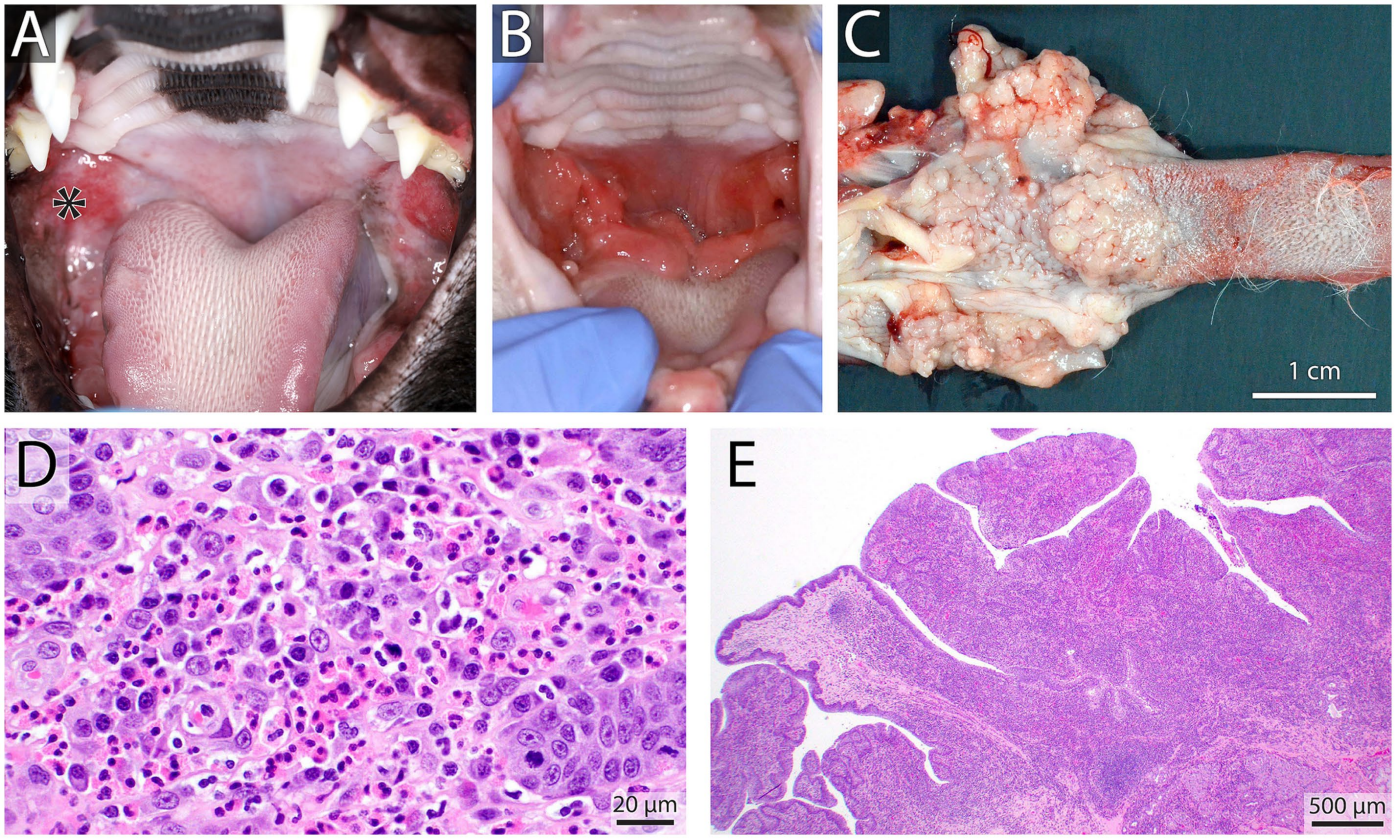
Caudal stomatitis in a 2-year-old female spayed domestic short hair cat noted on oral examination **(A)**, the patient tested positive for calicivirus on an oral swab and the inflammation was self-limiting. In contrast, shown in **(B)** is a 10-year-old male castrated domestic short hair cat with refractory proliferative caudal stomatitis involving the palatoglossal folds after full mouth extractions. Gross necropsy findings of the same cat highlight the nodular and hyperplastic appearance of the caudal tongue and palatoglossal folds **(C)**. Histopathology showed eosinophilic and plasmacytic gingivostomatitis **(D)** (H&E Bar = 20 μm) with significant papillary mucosal hyperplasia **(E)** H&E Bar = 500 μm.

Feline calicivirus (FCV) is a highly contagious virus that is a common cause of upper respiratory tract infections and oral disease in cats. Outdoor cats and cats in shelters are more likely to contract the virus as it is transmitted via direct contact with saliva, respiratory secretions, and fomites (48). FCV is known to cause ulcerative lesions in the oral cavity, specifically the tongue, by inducing necrosis of the epithelial cells (49). FCV’s role in feline chronic gingivostomatitis (FCGS) is widely accepted, but a direct causal relationship has not been definitively established (50). FCGS is diagnosed based on examination of the oral cavity and distribution of lesions, which include oral pain, halitosis, and bilateral inflammation to ulceration of the gingiva, alveolar, buccal, and caudal oral mucosa, lateral to the palatoglossal arches (50, 51) (Figure 4). The diagnosis can be confirmed histologically in the lesions with feline calicivirus immunohistochemical staining using a monoclonal antibody to FCV (52). The definitive association between FCV and FCGS is difficult to demonstrate because many cats with FCV will not develop any oral lesions or may have acute manifestations with a resolution of clinical signs and lesions within a month.

### Feline retroviruses

Feline immunodeficiency virus (FIV) and feline leukemia virus (FeLV) are retroviruses that are endemic worldwide in domestic cats. Transmission of FIV occurs primarily through bite wounds from infected cats and can lead to immunodeficiency in some patients. The feline leukemia virus is spread via saliva and other secretions and may be passed from queen to offspring. Infection may result in neoplasia, myelosuppression, and immunosuppression (53). FIV and FeLV are both reported to be associated with periodontitis and stomatitis (53). Specifically, retrovirus seropositivity rates are significantly higher in cats with oral cavity inflammation than in cats with healthy oral cavities (53).

Furthermore, FIV has been found to be significantly associated with gingivitis and feline resorptive lesions compared to virus-free cats (54). It is critical to determine the viral status of cats diagnosed with FCGS as viral status negatively affects prognosis for improvement following dental extractions; cats with FeLV were 7.5 times more likely to have no improvement after extractions than virus-free cats (55). In the same study, cats with lingual ulcers were 2.7 times more likely to have a decreased response to dental extractions (55). In cats with FCGS, knowing the viral status will allow clinicians to inform clients of potential outcomes following dental extraction more accurately and prepare them for the increased likelihood of needing long-term medical management.

### Bartonella

Bartonella is an intracellular bacteria transmitted by fleas, sandflies, or ticks to the cat. It is zoonotic and the causative agent of cat scratch fever in humans. Bartonella has been implicated in stomatitis and gingivitis, but study results have not consistently linked *Bartonella* seropositivity with gingivostomatitis in cats (51, 56). Currently, *Bartonella* is not widely believed to play a significant role in stomatitis in cats, and more research is warranted. Western blot is the diagnostic test of choice, and screening cats with stomatitis for *Bartonella* may be warranted, given its zoonotic potential.

### Canine papilloma virus

**Figure 5:**
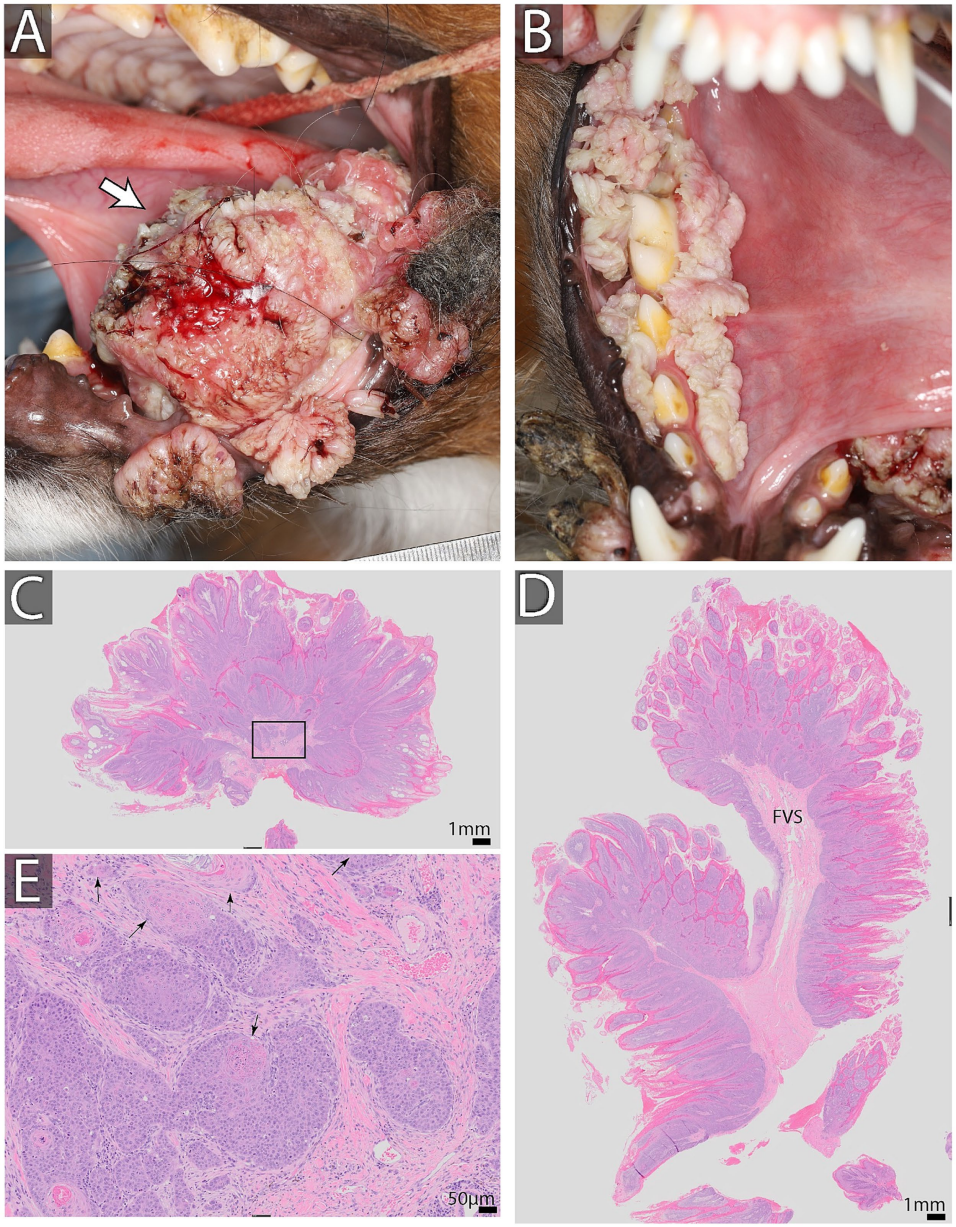
Oral papillomas and oral squamous cell carcinoma in a 2-year-old male neutered Australian shepherd dog found on the lingual aspect of the left mandibular second molar tooth **(A)**. Multiple papillomatous lesions were found surrounding the right mandibular dentition and at mucocutaneous junctions **(B)**. Biopsy results are shown in **(C–E)**. **(C,D)** Show low magnification of the biopsy stained with H&E. Note exuberant epithelial proliferation thrown into papillary projections supported by fibrovascular stroma (FVS). Bar = 1 mm. **(E)** High magnification of an area enclosed in a black rectangle in **C**. Note distinct epithelial islands demonstrating dysplastic changes. Specifically, the basilar epithelial layer is missing, and cells keratinize at the basal location (black arrows) Bar = 50 μm. This dysplasia is indicative of neoplastic transformation into squamous cell carcinoma. On additional sections of the neoplasm and associated mandible, it was diagnosed as a papillary type of squamous cell carcinoma.

Several species-specific types of *Papillomaviruses* infect dogs and cats. Papillomaviruses infect keratinocytes in both the haired skin and mucosa. Most infections are subclinical but may result in epithelial cell proliferation. The role of papillomavirus in the development of papillary squamous cell carcinoma has been debated in dogs and implicated in cancerogenesis in humans (57). There are currently 20 known types of canine papillomavirus (CPV). CPV3 and CPV16 strains, both Chipapillomaviruses, have been implicated in the development of squamous cell carcinoma in dogs (58). CPV-1 was once considered relatively benign, but one study demonstrated the malignant transformation of oral papillomas caused by CPV-1 into squamous cell carcinomas in multiple dogs (58). Oral manifestations of CPV include wart-like growths on mucosal surfaces in the oral cavity and the lesions can be localized anywhere from the lip margins to the pharynx. In the case of malignant transformation, there may be an invasion into the bone, leading to loss of teeth and a more considerable mass-like proliferation of tissue (Figure 5). Oral biopsies and imaging with dental radiographs and computed tomography are essential in patients with non-self-limiting or persistent lesions, especially if the lesions appear atypical for benign papillomas.

### Canine distemper virus

**Figure 6:**
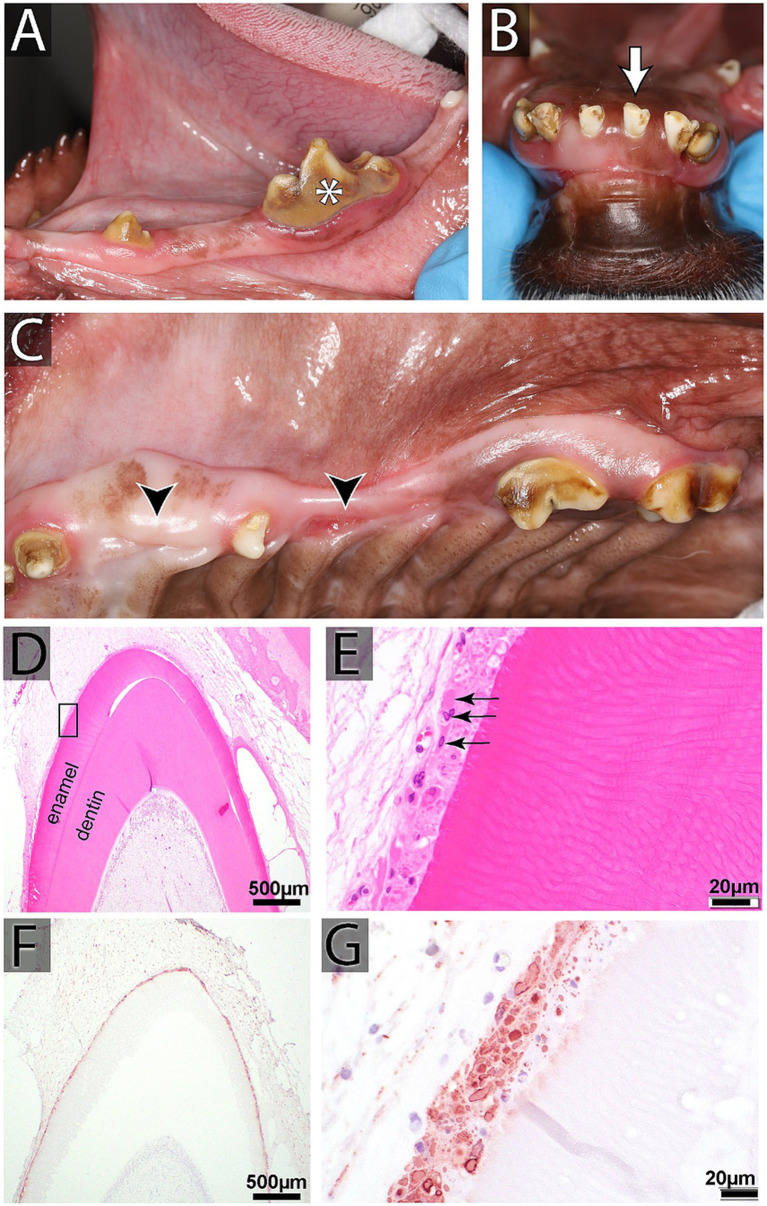
**(A–C)** Oral findings in a 2-year-old female spayed mixed breed dog affected by distemper, including intrinsic staining of multiple teeth, enamel hypoplasia, generalized odontodysplasia, and multiple missing and unerupted teeth. **(D,E)** Histopathology of developing teeth from another canine distemper virus (CDV)-affected dog (H&E). **(D)** Low magnification of a developing (unerupted) tooth at the bell stage with active production of enamel and dentin layers (Bar = 500 μm). **(E)** High magnification of the black rectangle in **D**, showing disruption of the typical palisading arrangement of ameloblasts underneath the enamel. Instead, the ameloblasts demonstrate severe cytopathic changes and have bright pink intranuclear and cytoplasmic inclusions (black arrows; Bar = 20 μm). **(F,G)** CDV immunohistochemistry staining of another developing tooth. **(F)** An intense immunoreactivity of the ameloblast layer is visible at low magnification (Bar = 500 μm). **(G)** Ameloblasts show strongly immunoreactive for CDV antigen at high magnification (Bar = 20 μm).

Canine distemper virus (CDV) is a *Morbillivirus* that affects dogs and is spread through oronasal contact via secretions and aerosol transmission. Infection in puppies prior to the eruption of permanent dentition typically leads to damage to the tooth roots, enamel, and dentin (59). Diffuse enamel hypoplasia typically results from a systemic infection, like CDV, resulting in a high fever and direct infection of the ameloblasts during enamel production. In dogs, hard tissue formation of permanent teeth occurs between birth and 8 weeks of age (60). Possible presentations include pits, fissures, and, the most commonly seen with CDV, enamel hypoplasia of the entire circumference of the crown (60). Enamel hypoplasia results in thinner than normal enamel that may appear stained tan to dark brown; an exposed area of dentin may be present, and there is often increased plaque retention due to the rough surface of the tooth (61) (Figure 6). These patients may also have missing and impacted teeth (62). All patients exhibiting enamel hypoplasia should receive cone beam CT or dental radiographs and charting. Missing teeth and malformed teeth also warrant cone beam CT or dental radiographs to detect unerupted teeth or evidence of endodontic disease.

### Fungal cryptococcosis in cats

Cryptococcosis is the most common systemic fungal infection in cats. Inhalation of spores from the environment allows the fungus to colonize the nasal passages of susceptible cats and can cause severe rhinitis (59). The nose is the most commonly affected part of the body in infected cats, with nasal discharge and facial swelling often noted on physical examination. Ulcers in the oral cavity and pharynx may also be noted along with proliferative lesions (63). Lesions on the tongue or gingiva may suggest a hematogenous spread of the infection (64). In one case report, the cat presented with oral bleeding from a proliferative and friable mass on the buccal and palatal aspect of the right maxilla. This mass closely resembled a squamous cell carcinoma in clinical presentation. However, histopathological evaluation was consistent with a fungal infection, and Cryptococcus was confirmed with PCR. Additionally, ulcerations were found on the palate of this cat, and on radiographs, there was loss of the periodontal ligament space and alveolar bone under the soft tissue proliferation (65). Diagnosis of cryptococcus in cats can be obtained with a rapid agglutination test on serum or culture of biopsied tissue from the nasal or oral cavity. A thorough fundic examination should be performed in any patient with oral manifestations of disease, especially in patients with suspected fungal disease, as associated chorioretinal lesions can commonly be present in these patients. With early detection and appropriate treatment, the prognosis for cryptococcosis is good (63).

### Mucocutaneous pyoderma

Mucocutaneous pyoderma (MCP) is a bacterial infection that can affect mucocutaneous junctions around the mouth, eyes, nose, genitals, and anus in both dogs and cats. The only way to confirm the diagnosis is by response to antibacterial therapy. Clinically, the lesions manifest as erythema, with erosions, depigmentation, and crusting. Histologically, the inflammation is concentrated in the superficial dermis and can be pleomorphic but classically predominated by plasma cells. MCP should not have evidence of basal cell degeneration, but this cellular change may occur due to inflammation, making the distinction from DLE more challenging (25).

## Oral manifestations of hematological disease

### Von Willebrand’s disease

**Figure 7:**
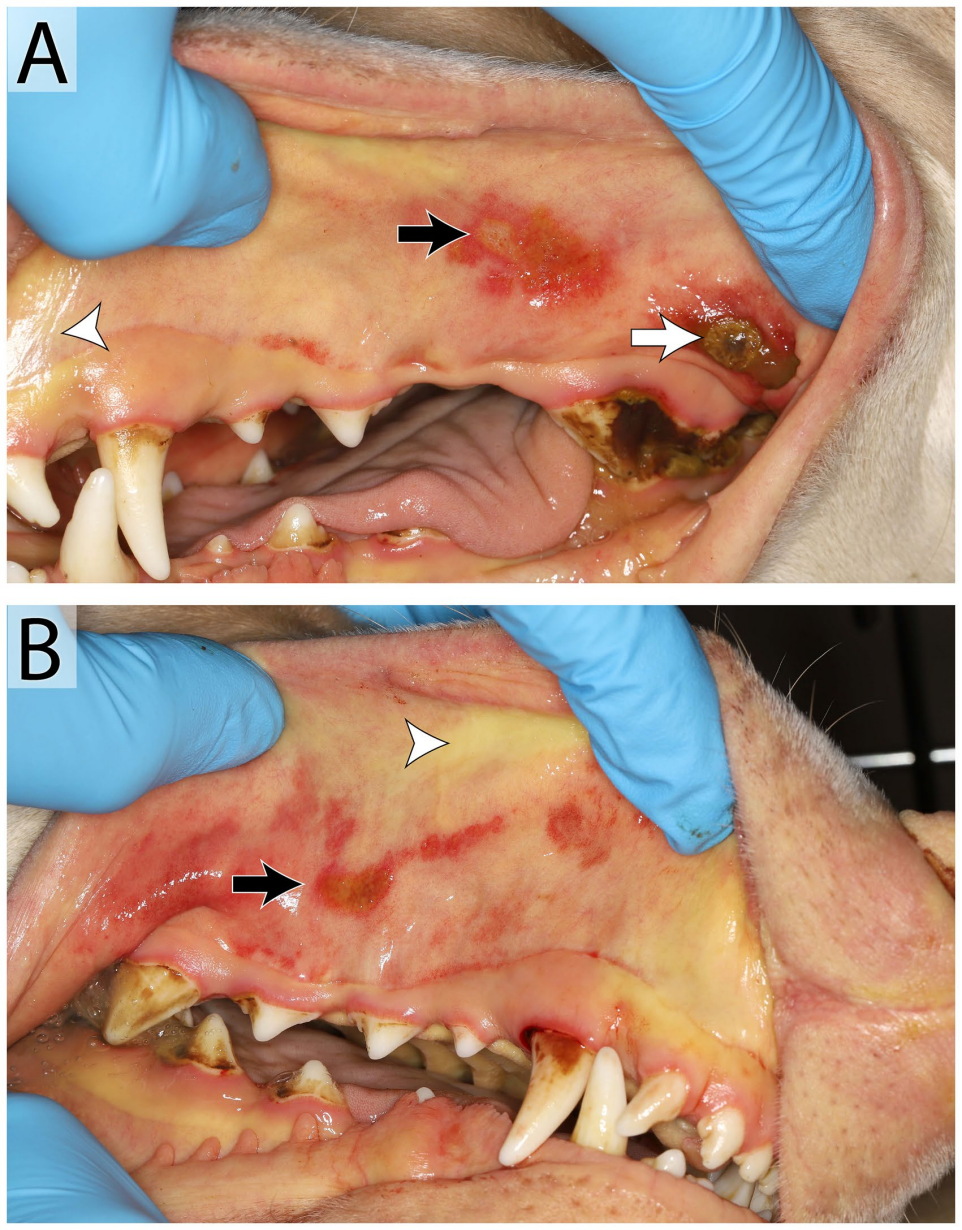
Manifestation of Von Willebrand’s disease in a 3-year-old male intact Doberman Pinscher. Oral examination **(A,B)** revealed petechia and ecchymoses (black arrow) and ulceration (white arrow) of the oral mucous membranes and wide-spread ecchymoses on the skin. Mucous membranes are icteric diffusely (white arrow head). The patient tested positive for von Willebrand’s Disease. Oral petechia are an uncommon finding with vWD and the bruising was likely a result of thrombocytopenia secondary to platelet consumption or immune-mediated destruction. The icterus noted in the oral cavity was the result of hyperbilirubinemia secondary to either hepatic dysfunction or, more likely, immune-mediated hemolytic anemia. Bilateral ulcerations on the buccal mucosa and under the tongue progressed. This may have been immune-mediated or the results of uremia, as this patient developed an acute kidney injury prior to euthanasia.

Von Willebrand’s disease (vWD) is the most common heritable bleeding disorder in dogs and is caused by a deficiency in von Willebrand’s Factor (vWF) (66). vWF is a glycoprotein in the endothelium that plays an essential role in primary hemostasis and platelet adhesion during blood clot formation. Three types of vWD exist, with Type 1 (normal protein structure but variably low levels) being the most common. Doberman pinschers, German shepherd dogs, and Golden retrievers are some of the breeds that are over-represented (66). Clinical signs range from absent to severe. Type II vWD is severe and has only been noted in German shorthair pointer dogs. Type III is uncommon and severe as there is a complete or near complete absence of vWF; Scottish terriers and Chesapeake Bay retrievers are overrepresented (67). Dogs with a deficiency may exhibit gingival bleeding, prolonged bleeding during oral surgery, and petechia and/or ecchymoses (67) (Figure 7).

### Immune-mediated hemolytic anemia and immune-mediated thrombocytopenia

Immune mediated hemolytic anemia (IMHA) is caused by the destruction of circulating red blood cells by the animal’s immune system and is one of the most common causes of anemia in small animals (68). IgG or IgM antibodies binding to the erythrocytes leads to immune destruction. There are many known underlying disorders and triggers for IMHA, including medications (sulfonamides, vaccines), neoplasia, infectious agents (viral, parasitic, and bacterial), and genetic predisposition (American cocker spaniel) (69). Oral symptoms associated with IMHA in dogs and cats include pallor and icteric mucous membranes. Immune-mediated thrombocytopenia is caused by immune destruction of circulating platelets by macrophages. Immune mediated thrombocytopenia (ITP) may be idiopathic or secondary to neoplasia, infectious disease, or medications (70). An increased risk of spontaneous hemorrhage occurs when the platelet count is <30,000 cells/μL (71). Oral lesions include petechia and ecchymoses on mucous membranes, pallor, and gingival bleeding that occur when platelet function or the total number in the bloodstream is affected.

### Vitamin K deficiency

Vitamin K is a fat-soluble vitamin found in green leafy vegetables, meat, eggs, and milk. It is also synthesized by microflora in the GI tract (72). The daily requirement is relatively small, and the liver is responsible for storing a few days’ worth of Vitamin K. Vitamin K is required to activate coagulation factors II, VII, IX, and X (72, 73), and deficiency results in an inability to clot the blood and subsequent hemorrhage. Anticoagulant rodenticide ingestion is in the top 10 toxicities seen in dogs (74). This toxin inhibits the recycling of vitamin K, resulting in hemorrhage within 3–5 days when the body runs out of activated coagulation factors (73). In one retrospective study of clinical bleeding in dogs that were exposed to anticoagulant rodenticide, 73% of dogs had evidence of cutaneous or mucosal hemorrhage, and 13% of dogs had bleeding in the oral cavity (73). Oral signs associated with Vitamin K deficiency include oral hemorrhage and pale mucous membranes. Other causes of deficiency include liver disease and nutritional deficiency, though this is rare considering most dogs are fed a commercial diet.

## Oral manifestations of miscellaneous systemic disorders

### Lymphoma

**Figure 8:**
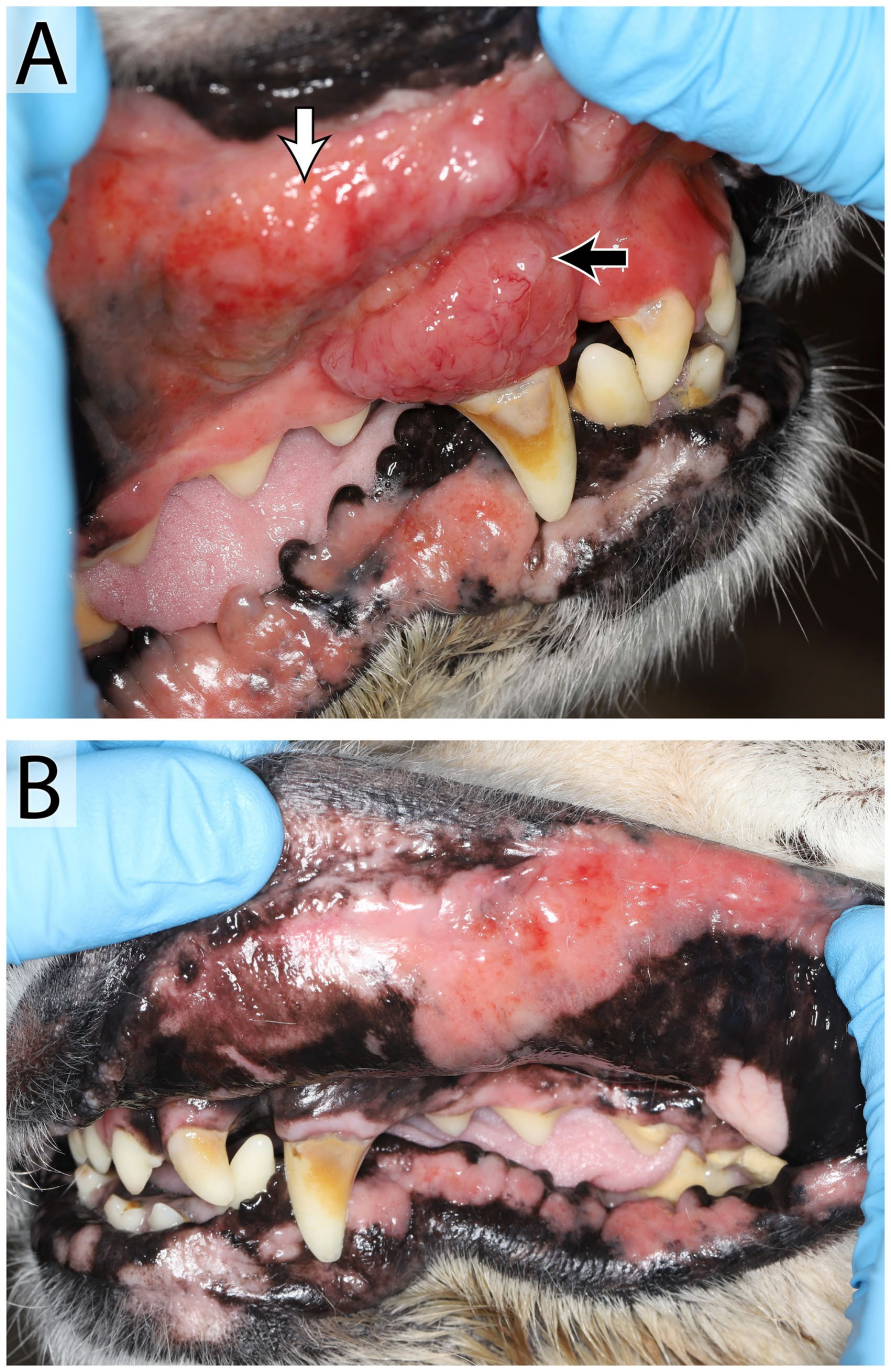
Oral lymphoma in a 14 year old female spayed Golden retriever that presented with an oral mass, hypersalivation, mild oral bleeding and discomfort. An irregular, exophytic, lobulated oral mass **(A)** was found at the level of the right maxillary canine tooth (black arrow), the adjacent mucosa and upper lip were irregular and firm (white arrow). Mucocutaneous junctions of the lips were erythematous bilaterally **(B)**. Right and left mandibular lymph nodes were enlarged and firm. Results of fine needle aspirate and cytology were consistent with large cell lymphoma in the right lymph node and lymphoid hyperplasia in the left. Mulitple biopsies of the oral mass and mucocutaneous junctions were recommended, further diagnostics were declined by the owner in favor of palliative care.

While lymphoma is one of the most common cancers in dogs, it is infrequently diagnosed in the oral cavity. In the rare cases when lymphoma does affect the oral cavity, clinical presentation may range from a single tumor to diffuse ulceration, hypopigmentation, and erythema (Figure 8). Horizontal alveolar bone loss, with near complete loss of alveolar bone, may be seen on radiographs in some cases (75). Cutaneous epitheliotropic T-cell lymphoma is an uncommon cancer seen in dogs with a highly variable clinical presentation. Lesions are standard on the skin but may also be present at mucocutaneous junctions and in the oral cavity in up to 50% of cases (76). Oral manifestations of epitheliotropic T-cell lymphoma include erosion and hemorrhage of oral mucous membranes, and lesions affecting the gingiva, tongue, and hard and soft palate (27, 76). The clinical presentation may be similar to autoimmune diseases that affect the oral cavity; thus, lymph node aspiration and cytology, biopsy, and histopathology are recommended for differentiation and prognostication.

### Adverse drug reactions

Gingival hyperplasia is the benign overgrowth of gingival tissue in response to trauma, inflammation, certain medications, or idiopathic causes. The medications reported to cause gingival hyperplasia include immunosuppressants (cyclosporin), calcium channel blockers (amlodipine, diltiazem), and anti-seizure medications (phenytoin) (77, 78). Enlargement of the gingiva typically begins within a few months of starting medication and can be widespread throughout the oral cavity. Overgrowth of the gingiva may sometimes be relieved by adjusting the dosage or discontinuing the medication when possible (79). Medical management with azithromycin was shown to be successful in treating gingival hyperplasia secondary to cyclosporin administration in one case series (79). Otherwise, periodic periodontal treatment and gingivoplasty may be required to control the gingival hyperplasia. A biopsy of the gingival tissue is needed for a definitive diagnosis to rule out other causes of gingival enlargement. Histologically, with gingival enlargement due to cyclosporine, a band of lymphoplasmacytic infiltration in the superficial dermis is accompanied by dermal edema and fibroplasia. The gingival epithelium can be hyperplastic to eroded with prominent rete pegs and individual apoptotic keratinocytes without satellitosis. In contrast, enlargement of the gingiva as a result of other medications is predominantly fibrotic (80).

Xerostomia, or dry mouth, may be caused by commonly prescribed medications, including beta-blockers, tricyclic antidepressants, benzodiazepines, and antihistamines (81). Xerostomia is known to cause halitosis and periodontal disease in humans (82, 83).

Osteonecrosis of the jaw (ONJ) is characterized by chronically exposed and necrotic bone of the maxilla and mandible. ONJ can further be divided into osteoradionecrosis of the jaw (ORNJ) and bisphosphonate-associated osteonecrosis of the jaw (BRONJ) or medication-related osteonecrosis of the jaw (MRONJ). A history of radiation for the treatment of oral tumors is a risk factor for developing necrosis of the jaw bones (84). Administration of bisphosphonate medications, like zoledronate or anti-angiogenic agents, is associated with the development of jaw osteonecrosis in humans, dogs and cats (85–87). Regardless of the cause, ONJ can lead to exposed necrotic bone in the mouth, draining tracts, swelling, halitosis, oral pain, and dysphagia (84, 85, 88).

### Nutritional deficiencies

Maintaining good oral health in animals requires a balanced diet. Gingivitis and periodontitis have been linked to and periodontal disesase has been associated with nutritional deficiencies of vitamins A, B, C, D and E (89). There is a likely decrease in the incidence of malnutrition among domesticated dogs and cats with the availability of nutritionally balanced commercial pet food diets.

## Discussion

Lesions in the oral cavity are common in veterinary medicine, and definitive diagnosis and treatment can be difficult. Differentiating primary dental disease and oral lesions stemming from systemic illness may be challenging. Oral manifestation of a systemic disorder should always be considered, especially when other systemic abnormalities are present. This review aims to help familiarize veterinarians with lesions that may be found in the oral cavity due to systemic disorders and to consider these findings when developing diagnostic plans for patients. The location of oral lesions, tissue types involved, and systemic clinical signs will help guide the next diagnostic steps. Targeted diagnostic modalities should be determined based on clinical presentation, oral examination, complete physical examination, and laboratory findings. These may include a urinalysis, coagulation panel, culture and sensitivity, fungal testing or PCR, and biopsies for histopathological analysis. Patients exhibiting petechia or ecchymoses on the oral mucous membranes and thrombocytopenia may require coagulation parameter testing and coagulopathies should be managed before any surgical intervention. A conventional or cone beam CT scan may be indicated when there is jaw swelling, a mass in the oral cavity, or evidence of necrosis.

When vesiculobullous and ulcerative lesions are found in the oral cavity and at the mucocutaneous junctions, neoplasia, immune-mediated, and autoimmune diseases should be considered, especially with concurrent skin involvement (25). While the appearance and distribution of lesions can aid a veterinarian in determining the cause, a biopsy is always required for a definitive diagnosis. Identifying the tissue layer and type of cells that are present will assist in identifying the disease process. The blisters and vesicles in the oral cavity are fragile and often rupture quickly, leaving deep ulcerations and erosions with the risk of loss of pathognomonic features like tombstone cells in pemphigus vulgaris. Thus, the strategy while obtaining samples for biopsy should include multiple locations, including healthy looking tissues, with the concurrent delicate handling of tissues. In addition, cats with inflammatory lesions in the mouth, including severe gingivitis and stomatitis, should be screened for infectious diseases as part of the baseline diagnostic plan. While infectious agents may not directly cause oral disease, they likely play a role in the disease. Regarding the canine papillomavirus, further research is necessary to determine if persistent papillomatous lesions or malignant transformation is due to the CPV strain or a compromise of the immune system.

Limitations of this narrative review include limited research in the veterinary literature focusing on the oral manifestations of systemic disease, particularly metabolic and endocrine diseases. In comparison, entire textbooks are dedicated to the link between systemic disorders and disease in the oral cavity in humans (90). In the veterinary field, extrapolation of that information often occurs.

## Conclusion

Oral manifestations of systemic disorders may be encountered in dogs and cats. Recognition, diagnosis, and treatment often require a multidisciplinary approach. To determine an appropriate treatment plan, assessing the patient’s history, physical examination findings, and diagnostic results is prudent to establish a clinical-pathologic correlation. Finally, more research is needed to focus on the oral manifestations of systemic disorders in cats and dogs.
